# Designing a High-Throughput Somatic Mutation Profiling Panel Specifically for Gynaecological Cancers

**DOI:** 10.1371/journal.pone.0093451

**Published:** 2014-03-26

**Authors:** Vivian M. Spaans, Marjolijn D. Trietsch, Stijn Crobach, Ellen Stelloo, Dennis Kremer, Elisabeth M. Osse, Natalja T. ter. Haar, Ronald van Eijk, Susanne Muller, Tom van Wezel, J. Baptist Trimbos, Tjalling Bosse, Vincent T. H. B. M. Smit, Gert Jan Fleuren

**Affiliations:** 1 Department of Pathology, Leiden University Medical Center, Leiden, The Netherlands; 2 Department of Gynaecology, Leiden University Medical Center, Leiden, The Netherlands; 3 Department of Molecular Epidemiology, Leiden University Medical Center, Leiden, The Netherlands; 4 Sequenom GmbH, Hamburg, Germany; University of Navarra, Spain

## Abstract

Somatic mutations play a major role in tumour initiation and progression. The mutation status of a tumour may predict prognosis and guide targeted therapies. The majority of techniques to study oncogenic mutations require high quality and quantity DNA or are analytically challenging. Mass-spectrometry based mutation analysis however is a relatively simple and high-throughput method suitable for formalin-fixed, paraffin-embedded (FFPE) tumour material. Targeted gene panels using this technique have been developed for several types of cancer. These current cancer hotspot panels are not focussed on the genes that are most relevant in gynaecological cancers. In this study, we report the design and validation of a novel, mass-spectrometry based panel specifically for gynaecological malignancies and present the frequencies of detected mutations. Using frequency data from the online Catalogue of Somatic Mutations in Cancer, we selected 171 somatic hotspot mutations in the 13 most important genes for gynaecological cancers, being *BRAF, CDKN2A, CTNNB1, FBXW7, FGFR2, FGFR3, FOXL2, HRAS, KRAS, NRAS, PIK3CA, PPP2R1A* and *PTEN*. A total of 546 tumours (205 cervical, 227 endometrial, 89 ovarian, and 25 vulvar carcinomas) were used to test and validate our panel, and to study the prevalence and spectrum of somatic mutations in these types of cancer. The results were validated by testing duplicate samples and by allele-specific qPCR. The panel presented here using mass-spectrometry shows to be reproducible and high-throughput, and is usefull in FFPE material of low quality and quantity. It provides new possibilities for studying large numbers of gynaecological tumour samples in daily practice, and could be useful in guided therapy selection.

## Introduction

Cancer genomes carry somatic mutations, and the mutation spectrum varies by tumour type and subtype [1], [2]. Evaluating a broad range of key cancer gene mutations across diverse cancers has the potential for identifying clinically relevant mutations. Studies of melanoma, lung, colorectal, and breast carcinomas have shown that the somatic mutation status can be used to predict prognosis and guide tumour-specific treatment strategies [3]–[6]. Gynaecological malignancies represent 15–20% of all new cancer cases in women worldwide, and numbers continue to increase [7], but the carcinogenesis of gynaecological malignancies is diverse and the role of somatic mutations is not yet fully elucidated [1].

Over the last decade, somatic mutations and their role in targeted therapy have been studied in gynaecological malignancies, but not yet to the same extent as in other types of cancer such as breast and colon cancer. Mutation profiling of gynaecological malignancies may identify novel drug targets and help predict patient prognosis and tumour response to treatment. Research has revealed overlapping genetic changes as well similar affected signalling pathways in the different types of gynaecological tumours [8]–[14].

When studying large numbers of patient material, we face two types of problems: technical applicability and tumour specificity. Nowadays, only a limited number of genes is screened in clinical practice. It is expected that this number will increase considerably in the near future. Therefore, a fast and trustworthy method to detect mutations is required. This technique must be suitable for DNA extracted from formalin fixed paraffin embedded (FFPE) tissue, which is often of low quality, or from small tissue biopsies, which is of low quantity. Matrix-assisted laser desorption/ionization time-of-flight mass spectrometry (MALDI-TOF) has proved to meet all these criteria [15]–[17].

As for tumour specificity, currently, several oncogene panels based on different techniques are (commercially) available. These panels have been successfully used in studying large amounts of tumour samples, in order to draw the landscapes of somatic mutations that characterise tumour types [18]–[22]. A selection of genes and mutations relevant to tumour subtypes has successfully led to the design of tumour specific panels [15], [16], [23]. As yet, there are no panels available that are specifically designed to target gynaecological tumours. Therefore, we aimed to develop a high-throughput mutation panel specified for gynaecological malignancies.

A meta-analysis of the COSMIC (Catalogue of Somatic Mutations in Cancer) online database [24], was performed to design a MALDI-TOF-based, high-throughput mutation panel that covers somatic mutations in 13 genes that are most frequently reported to be involved in gynaecological malignancies. We tested and validated this panel in a set of 546 cervical, endometrial, ovarian and vulvar carcinoma samples. Here, we present the design of a gynaecological cancer specific panel and the frequencies of somatic mutations identified using it.

## Materials and Methods

All human tissue samples in this study were used according to the medical ethical guidelines described in the Code for Proper Secondary Use of Human Tissue established by the Dutch Federation of Medical Sciences (www.federa.org, an English translation of the Code can be found here:

http://www.federa.org/sites/default/files/digital_version_first_part_code_of_conduct_in_uk_2011_12092012.pdf).

Patients receive information on the secondary use of tissue that is sampled for diagnostic use. They can actively object to secondary use. Accordingly to these guidelines, all human material used in this study has been anonymized. Because of this anonymization procedure, retrospective research does not require ethical approval from the Institutional Review Board and individual patients' permission is not needed.

### Panel design

First, PubMed and COSMIC [24] searches were performed to select genes and mutations for inclusion in the gynaecologic-specific mutation panel. Selection was based on whether a mutation was repeatedly found to be mutated in gynaecological malignancies. Second, in order to cover a high percentage of the reported variants per gene, the most frequent mutations were selected to obtain a fair gynaecological-tissue-specific coverage, as only hotspot mutations were appropriate for analysis with the MALDI-TOF technique. We aimed to select genes in which for at least one of the studied gynaecological cancer types (e.g. vulvar, cervical, endometrial or ovarian cancer), at least 30% of all reported mutations occurred on less than 10 different sites on the gene.

### Establishing a gynaecologic specific ‘hotspot’ gene panel – GynCarta 1.0

Consulting PubMed and COSMIC databases clearly showed an overlap in top ten genes mutated in cervical, endometrial and ovarian cancer. Few somatic mutation studies have been performed on vulvar cancer and therefore for this tumour type we relied on frequencies found in similar tumour types (e.g. squamous cell carcinoma of the skin on other sites, and squamous cell carcinoma of head and neck). The most frequently mutated genes that met our inclusion criteria were selected for the panel. The first panel we designated ‘GynCarta 1.0’ (Sequenom, Hamburg, Germany) consisted of 89 assays (12 multiplexes) to detect 154 mutations in 12 genes that met our inclusion criteria: *BRAF, CDKN2A, CTNNB1, FBXW7, FGFR2, FGFR3, FOXL2, HRAS, KRAS, NRAS, PIK3CA*, and *PTEN*.

### Assay design

MySequenom.com online assay design tools were used to design the somatic mutation detection assays. A maximum multiplex level of 12 assays per well was applied. If possible, the mutant allele extension peaks were designed as first detected allele peaks and the wild type extension peaks as the last detected allele peaks to reduce the danger of false positives from salt adducts. All assays were validated on wild type DNA, negative controls and selected known positive mutation samples.

### Mutation detection

Mutation detection was performed at the Leiden University Medical Center following the manufacturer's protocol (Sequenom, Hamburg, Germany) as described previously [29]. Briefly, wild type and mutant DNA was amplified by multiplex PCR. Shrimp alkaline phosphatase treatment inactivated surplus nucleotides. A primer extension reaction (iPLEX Pro) was performed with mass-modified terminator nucleotides, and the product was spotted on a SpectroCHIP (Sequenom, Hamburg, Germany). Mutant and wild type alleles were then discriminated using MALDI-TOF mass spectrometry.

### Data analysis

Data were analysed with MassARRAY Typer Analyser software (TYPER 4.0.22, Sequenom, Hamburg, Germany). Mutations were detected by a minimum 5% threshold of the mutant allele peak. Three investigators blinded to tumour identification manually reviewed the output, and a consensus determination was reached. Statistical analyses were performed with IBM SPSS statistics Data Editor version 20.0. The independent Students *t*-test was used to compare baseline variables, and Fisher's exact test was used to analyse categorical and normally distributed numerical data. *P*-values ≤0.05, corresponding to 95% confidence intervals, were considered statistically significant. All tests were two-tailed.

### Samples

First, a training set of 51 FFPE samples (26 cervical, 17 endometrial, 6 ovarian and 2 vulvar cancer samples) was used to test the efficacy of the designed panel. After minor technical adjustments and improvements of the panel, the number of patients for each tissue type was extended.

In total, DNA from 548 tumour samples from cervical (*N = *209), endometrial (*N = *227), ovarian (*N = *89), and vulvar (*N = *25) carcinoma patients was isolated. Two cervical cancer samples failed for all mass spectrometry assays and were excluded from further analyses. The following baseline parameters were collected: age, FIGO (International Federation of Gynaecology and Obstetrics) stage, histopathological diagnosis, tumour grade if applicable, and human papillomavirus (HPV) positivity and type in cases of cervical and vulvar tumours (Table 1).

**Table 1:** Baseline characteristics.

		Cervical	Endometrial	Ovarian	Vulvar
		carcinomas	carcinomas	carcinomas	carcinomas
		*N* = 205	*N* = 227	*N* = 89	*N* = 25
Age, median (IQR)		43 (35–55)	69 (65–75)	62 (52–69)	74 (52–80)
FIGO stage, *N* (%)	I	159 (78)	210 (92)	16 (18)	6 (24)
	II	44 (21)	11 (5)	13 (15)	8 (32)
	III		4 (2)	39 (44)	7 (28)
	IV			9 (10)	3 (12)
	Unstaged	2 (1)	2 (1)	12 (14)	1 (4)
Histology, *N* (%)	Squamous cell carcinoma	166 (81)			25 (100)
	Adenocarcinoma	24 (12)			
	Adenosquamous carcinoma	15 (7)			
	Endometrioid adenocarcinoma		206 (90)	42 (47)	
	Serous adenocarcinoma		17 (7)	26 (29)	
	Mucinous adenocarcinoma		2 (1)	13 (15)	
	Clear cell adenocarcinoma		2 (1)	6 (7)	
	Mixed-type carcinoma			2 (2)	
Grade, *N* (%)	1–2	N.A.	179 (79)	52 (58)	N.A.
	3		49 (21)	30 (34)	
HPV, *N* (%)	Positive	186 (91)	N.A.	N.A.	6 (24)
	HPV16	117 (63)			5 (83)
	HPV18	42 (23)			1 (17)

The baseline characteristics for all 546 gynaecological malignancies included in this study. IQR  =  inter-quartile range; FIGO  =  International Federation of Gynaecology and Obstetrics; HPV  =  human papillomavirus; N.A.  =  not applicable.

### DNA isolation

DNA was isolated from FFPE tissue blocks for 505 samples and from fresh frozen (FF) tissue for 43 ovarian carcinomas. Three to five 0.6-mm diameter tissue cores of variable length were taken from the FFPE blocks from a selected area comprising ∼70% tumour cells. In 34 samples, tumour cells were diffusely distributed, and therefore micro-dissection was performed on 10 haematoxylin-stained 10-μm sections to obtain a high percentage of tumour cells. DNA isolation was performed as described before [25] followed by DNA purification (NucleoSpin Tissue kit, Machery-Nagel, Germany) or was performed fully-automated using the Tissue Preparation System (Siemens Healthcare Diagnostics, NY, USA) [28].

### DNA quality

DNA of all samples was isolated and tested for quality; 493 (90%) samples scored ≥1 for DNA quality using PCR, and this was considered sufficient. Two samples, both cervical carcinomas with a DNA quality score of 0, failed in all assays, giving a success rate of 99.99%. Both samples were excluded from further analysis. In general, samples with low quality DNA were more likely to fail in some assays, but 27 out of 48 samples (56%) with DNA quality scores of 0 were analysed successfully in all assays, and 40 out of 48 (83%) low quality samples were analysed successfully in more than 90% of the assays. The percentage of successfully analysed samples did not differ significantly between FFPE and fresh frozen samples. This confirms that the MALDI-TOF mutation detection method is highly suitable for the analysis of lower quality, FFPE-extracted DNA.

### Validation

**Figure 1:**
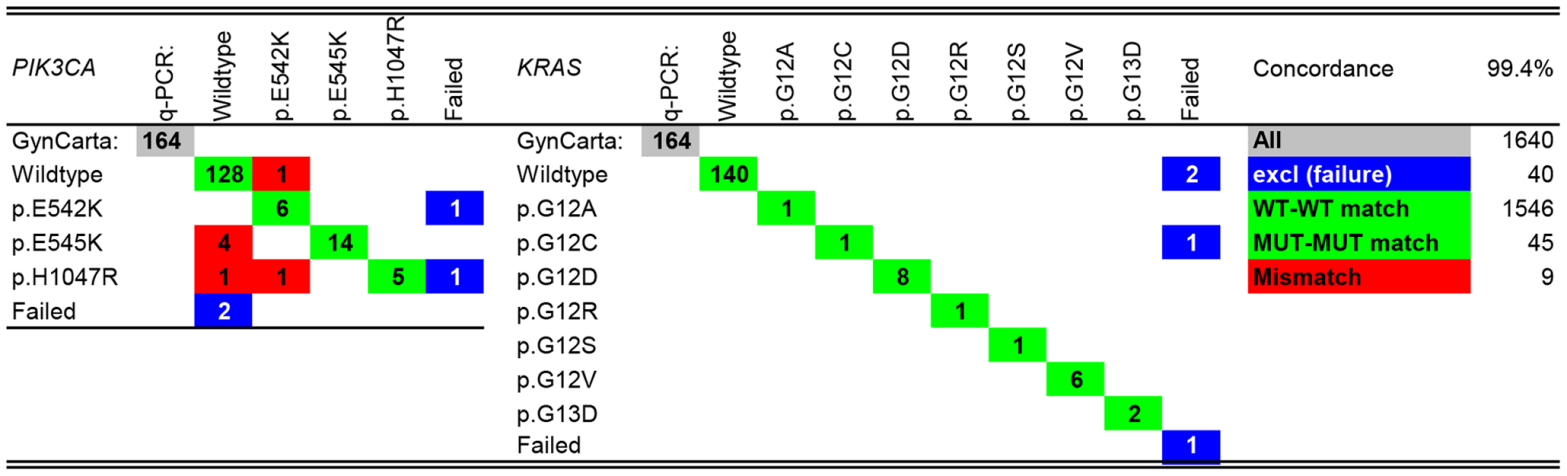
Concordance between MALDI-TOF mutation genotyping and allele-specific qPCR results. The concordance between MALDI-TOF mutation genotyping (GynCarta, Sequenom, Hamburg, Germany) and allele-specific qPCR for 3 *PIK3CA* and 7 *KRAS* mutations was determined for 164 (30% of the total cohort of 546 carcinomas) samples to validate the results. Concordance was calculated for all wild type-wild type matches (1546 in total) and all mutation-mutation matches (45 in total) in all reactions (164*10, 1640 in total). Failed reactions were excluded because comparison was not possible (4*3 for *PIK3CA* and 4*7 for *KRAS*; 40 in total). This lead to a concordance of (1546+45)/(1640−40)  = 0.994. WT  =  Wild type; MUT  =  mutant.

In total, 546 tumour samples were included in this study. To assess assay reproducibility, 57 (10%) samples were tested in duplicate and another 26 (5%) in triplicate. Of the initially detected mutations in these samples, 95% (40/42) was confirmed in duplicate and 97% (30/31) was confirmed in triplicate. Non-template (*N* = 4) and wild type leukocyte DNA (*N* = 2) controls were included in each multiplex to obtain negative and wild type MALDI-TOF spectra. Furthermore, for a random30% (163 samples), *KRAS* and *PIK3CA* mutations were validated using allele-specific qPCR as described previously [26] on 7 mutation variants of *KRAS* (p.G12C, p.G12R, p.G12S, p.G12V, p.G12A, p.G12D, p.G13D) and 3 mutation variants of *PIK3CA* (p.E542K, p.E545K, p.H1047R), and a concordance rate of 99.4% was attained. (figure 1) The GynCarta panel detected more mutations than allele specific qPCR did. This could be explained by the fact that mass spectrometry is able to detect mutant alleles with a lower frequency (down to 5%) than allele-specific PCR is (down to 20%). The fact that we did not find any mutations in the wild type control DNA, or in any of the H_2_O negative controls strengthens our belief that these additional mutations are true mutations rather than false positives.

### Improving the panel and creating GynCarta 2.0

With the first mutational data from GynCarta 1.0 and literature reports of new oncogenic mutations, we were able to improve the GynCarta 1.0 panel by removing assays of mutations that were not detected (*CDKN2A* D108Y, D108XA, Y108XC; *FGFR3* Y373C, A391E, K650Q, K650E, K650T, K650M, S371C; *KRAS* G13S and *NRAS* G13V, G13A, G13D, G13C, G13R, G13S) and by adding 10 new hotspot mutations of the already included genes. This way, the coverage of *FGFR2* and *PIK3CA* was increased from 59% to 71% and from 72% to 76%, respectively. Furthermore, assays that had shown to be difficult to interpret because of small artefact peaks were improved. During the testing and validation period, *PPP2R1A*, a new gene of interest, had emerged from the literature [27]–[29]. Nine mutations of this gene were also added to the panel, thus creating ‘GynCarta version 2.0’. A complete overview of the mutations included in the GynCarta 2.0 mutation panel is given in table 2, with the added assays listed in bold. The assays for GynCarta 2.0 were organised in such a way, that a total of 13 multiplexes could be used to analyse the full panel, concentrating the new assays on 4 multiplexes. These 4 multiplexes were used to analyse the 497 samples of the confirmation set.

**Table 2:** Design of GynCarta 2.0.

GENES (13)	*BRAF*	*CDKN2A*	*CTNNB1*	*FBXW7*	*FGFR2*	*FGFR3*	*FOXL2*	*HRAS*	*KRAS*	*NRAS*	*PIK3CA*	*PTEN*	*PPP2R1A*
Mutations	p.V600E	p.R58*	p.D32A	p.R465C	p.S252W	p.R248C	p.C134W	p.G12A	p.G12A	p.G12A	p.R88Q	p.K6fs*4	**p.P179L**
	p.V600K	p.R58X	p.D32G	p.R465H	**p.P253R**	p.S249C		p.G12C	p.G12C	p.G12C	p.E542K	p.E7*	**p.P179R**
	p.V600R	p.R80*	p.D32H	p.R479Q	**p.P253L**	p.G370C		p.G12D	p.G12D	p.G12D	p.E545A	p.F37S	**p.R183G**
	p.V600L	p.D108Y	p.D32N	p.R479L	p.Y375C	p.S371C		p.G12R	p.G12F	p.G12R	p.E545G	p.R84G	**p.R183W**
		p.D108A	p.D32V	p.R505C	**p.C382R**	p.Y373C		p.G12S	p.G12R	p.G12S	**p.E545D**	p.R130*	**p.R183Q**
		p.D108C	p.D32Y		**p.N549K**	p.A391E		p.G12V	p.G12S	p.G12V	p.E545K	p.R130fs*4	**p.S256F**
		p.W110*	p.S33A		**(T>A)**	p.K650E		p.G13C	p.G12V	p.G13A	p.Q546E	p.R130G	**p.S256Y**
		p.W110X	p.S33C		p.N549K	p.K650Q		p.G13D	p.G13A	p.G13C	p.Q546K	p.R130L	**p.W257C**
		p.P114L	p.S33F		(T>G)	p.G697C		p.G13R	p.G13C	p.G13D	**p.Q546R**	p.R130P	**p.R258H**
		p.P114X	p.S33P		**p.K659E**			p.G13S	p.G13D	p.G13R	**p.Q546P**	p.R130Q	
			p.S33Y					p.G13V	p.G13R	p.G13S	**p.Q546L**	p.R173C	
			p.G34E					p.G13X	p.G13V	p.G13V	p.Y1021C	p.R173H	
			p.G34R					p.Q61H	p.Q61E	p.Q61E	p.T1025A	p.Q214*	
			p.G34V					(C>A)	p.Q61H	p.Q61K	p.T1025X	p.R233*	
			p.S37A					p.Q61H2	(T>A)	p.Q61L	p.M1043I	p.R234W	
			p.S37C					(C>G)	p.Q61H	p.Q61P	(G>A)	p.P248fs*5	
			p.S37F					p.Q61K	(T>G)	p.Q61R	p.M1043I	p.C250fs*2	
			p.S37P					p.Q61L	p.Q61K		(G>T)	p.K267fs*9	
			p.S37T					p.Q61P	p.Q61L		**p.M1043V**	p.K267fs*31	
			p.S37Y					p.Q61R	p.Q61P		p.H1047L	p.V290fs*1	
			p.T41A						p.Q61R		p.H1047R	p.L318fs*2	
			p.T41I								p.H1047Y	p.T321fs*23	
			p.T41N									p.N323fs*2	
			p.T41S									p.N323fs*21	
			p.S45C									p.R335*	
			p.S45F										
			p.S45P										
			p.S45Y										
Total (171)	4	10	28	5	6	9	1	18	19	17	20	25	9
Assays (99)	2	5	12	4	5	8	1	8	7	6	13	22	6

The panel GynCarta 2.0, (Sequenom, Hamburg, Germany) consists of 13 multiplexes containing 99 assays to detect 171 mutations in 13 genes that are most frequently described to be involved in gynaecological malignancies according to a COSMIC meta-analysis. Assays that were added to create GynCarta 2.0 are depicted in bold.

## Results

### Mutations identified using GynCarta 1.0 and 2.0

Mutation genotyping using GynCarta 1.0 revealed 395 mutations in 273 (50%) samples. The most mutations were detected in endometrial carcinomas (177 samples (64%)), followed by ovarian carcinomas (33 samples (37%)), cervical carcinomas (67 samples (33%)), and vulvar carcinomas (5 samples (20%)). *PIK3CA* was mutated most frequently (122 samples), followed by *PTEN* (97 samples) and *KRAS* (64 samples). No mutations were found in *BRAF* and *FOXL2*.

Mutation genotyping using GynCarta 2.0 detected an additional 36 mutations: 4 on *FGFR2* and 5 on *PIK3CA*. *PPP2R1A* mutations were detected in 27 samples (7 cervical, 18 endometrial, 2 ovarian and 0 vulvar samples).

**Figure 2:**
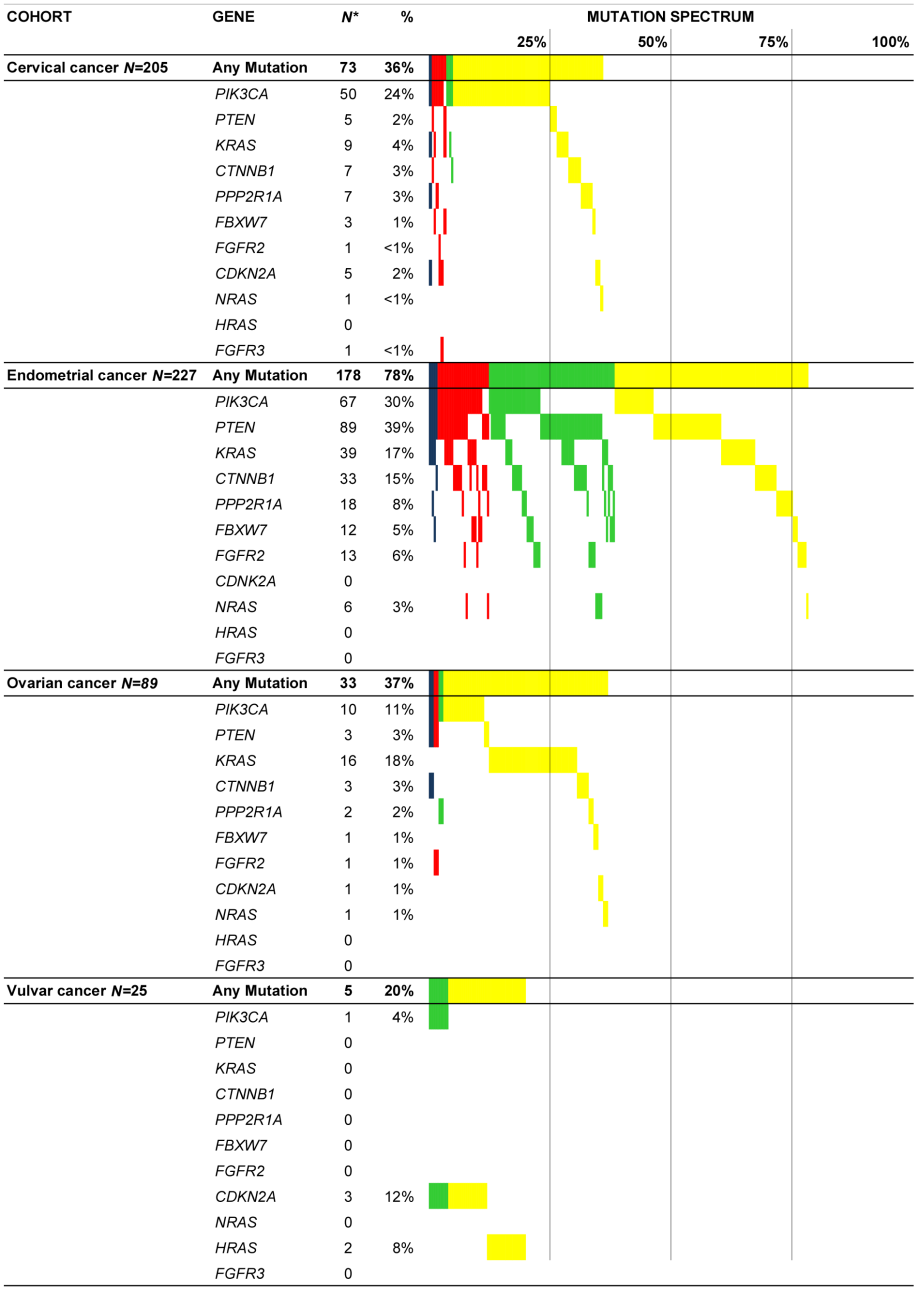
Mutation Spectrum. The spectrum and frequencies of mutations identified using MALDI-TOF in 546 gynaecological carcinomas. The mutation spectrum is shown (from top to bottom) for cervical (*N* = 205), endometrial (*N* = 227), ovarian (*N* = 89), and vulvar carcinomas (*N* = 25). From left to right, *N* is the number of samples with the mutation, ‘%’ is the percentage of mutated samples within the cohort, and bars represent the percentages graphically: blue, 4 mutations per sample (*N* = 6); red, 3 mutations per sample (*N = *29); green, 2 mutations per sample (*N = *65); and yellow, 1 mutation per sample (*N = *189).

Since panel version 1.0 and 2.0 had some overlapping assays, we were able to compare the results of both panels. We did not detect any discrepant mutation calls, but we were able to analyse assays that were hard to interpret in GynCarta 1.0 because these assays had improved in GynCarta 2.0. We also obtained successful output for 3 samples that had failed in GynCarta 1.0. The mutation frequencies for each locus are summarized in table 3. The mutation spectrum is visualised in figure 2.

**Table 3:** Mutation Frequencies as detected by GynCarta 2.0.

Tissue	CC^1^	EC^2^	OC^3^	VC^4^	Total	Tissue	CC	EC	OC	VC	Total
Gene	*N = *205	*N = *227	*N = 89*	*N = *25	*N = *546	Gene	*N = *205	*N = *227	*N = 89*	*N = *25	*N = *546
***PIK3CA^5^***	**50 (24)**	**67 (30)**	**10 (11)**	**1 (4)**	**128 (23)**	***CTNNB1^5^***	**7 (3)**	**33 (15)**	**3 (3)**	**0**	**43 (8)**
p.E545K	33	13	1	1	48/542	p.S37F	1	10	0	0	11/537
p.H1047R	2	13	5	0	20/542	p.S45F	1	5	0	0	6/543
p.E542K	15	3	1	0	19/542	p.G34R	2	1	0	0	3/537
p.R88Q	1	16	1	0	18/542	p.T41A	1	3	0	0	4/546
p.M1043I(T)	0	5	1	0	6/535	p.D32V	0	2	0	0	2/543
p.Q546R	0	3	1	0	4/468	p.D32Y	0	2	0	0	2/543
p.Y1021C	0	4	0	0	4/538	p.S33F	0	2	0	0	2/542
p.T1025A	0	4	0	0	4/530	p.D32N	1	1	0	0	2/543
p.H1047Y	0	3	0	0	3/541	p.S37C	0	1	1	0	2/537
p.E545A	0	2	0	0	2/542	p.T41I	1	0	0	0	1/542
p.Q546K	0	2	0	0	2/537	p.S37P	0	1	0	0	1/544
p.Q546L	0	1	0	0	1/468	p.D32H	0	1	0	0	1/543
p.M1043I(A)	0	1	0	0	1/535	p.S33A	0	1	0	0	1/544
p.M1043V	0	1	0	0	1/490	p.S33C	0	1	0	0	1/542
p.H1047L	0	1	0	0	1/542	p.S33Y	0	1	0	0	1/542
***PTEN^5^***	**5 (2)**	**89 (39)**	**3 (3)**	**0**	**97 (18)**	p.G34V	0	1	0	0	1/542
p.R130G	1	35	1	0	37/542	p.S45P	0	1	0	0	1/542
p.R130fs*4	0	19	2	0	21/545	p.G34E	0	0	1	0	1/542
p.L318fs*2	0	10	0	0	10/542	p.S37Y	0	0	1	0	1/537
p.R233*	0	7	0	0	7/543	***PPP2R1A^5^***	**7 (3)**	**18 (8)**	**2 (2)**	**0**	**27 (5)**
p.R130*	1	5	0	0	6/542	p.R258H	5	3	0	0	8/493
p.T323fs*2	0	5	0	0	5/542	p.R183W	1	6	0	0	7/490
p.R173C	0	4	0	0	4/540	p.P179L	0	5	0	0	5/490
p.R173H	0	2	1	0	3/539	p.P179R	2	1	1	0	4/490
p.E7*	0	3	0	0	3/545	p.R183Q	0	2	0	0	2/463
p.K267fs*31	1	2	0	0	3/542	p.S256F	0	1	1	0	2/463
p.R130L	0	1	0	0	1/544	***FBXW7***	**3 (1)**	**12 (5)**	**1 (1)**	**0**	**16 (3)**
p.R130P	0	1	0	0	1/544	p.R465H	2	6	1	0	9/536
p.R234W	1	1	0	0	2/495	p.R465C	1	3	0	0	4/540
p.K267fs*9	0	2	0	0	2/536	p.R505C	0	3	0	0	3/542
p.Q214*	1	1	0	0	2/544	***FGFR2***	**1 (<1)**	**13 (6)**	**1 (1)**	**0**	**15 (3)**
p.P248fs*5	0	1	0	0	1/545	p.S252W	0	9	1	0	10/533
p.V290fs*1	0	1	0	0	1/542	p.K659E	0	2	0	0	2/492
***KRAS***	**9 (4)**	**39 (17)**	**16 (18)**	**0**	**64 (12)**	p.N549K (A)	1	1	0	0	2/491
p.G12V	2	10	8	0	20/544	p.N549K (G)	0	1	0	0	1/541
p.G12D	4	13	3	0	20/544	***CDKN2A***	**5 (2)**	**0**	**1 (1)**	**3 (12)**	**9 (2)**
p.G13D	0	8	0	0	8/544	p.R58*	3	0	0	1	4/535
p.G12C	1	3	2	0	6/544	p.R80*	0	0	0	2	2/535
p.G12A	0	4	1	0	5/544	p.W110*	1	0	1	0	2/541
p.Q61H(G)	0	1	1	0	2/542	p.P114L	1	0	0	0	1/540
p.G12S	1	0	0	0	1/544	***NRAS***	**1 (<1)**	**6 (3)**	**1 (1)**	**0**	**8 (1)**
p.G12R	0	0	1	0	1/544	p.G12S	0	2	0	0	2/542
p.G13S	1	0	0	0	1/465	p.Q61L	0	2	0	0	2/541
						p.Q61K	0	1	1	0	2/541
						p.Q61R	1	0	0	0	1/541
						p.G12D	0	1	0	0	1/538
						***HRAS***	**0**	**0**	**0**	**2 (8)**	**2 (<1)**
						p.G12D	0	0	0	2	2/538
						***FGFR3***	**1 (<1)**	**0**	**0**	**0**	**1 (<1)**
						p.S249C	1	0	0	0	1/523
						***BRAF***	**0**	**0**	**0**	**0**	**0**
						***FOXL2***	**0**	**0**	**0**	**0**	**0**

1 Cervical, ^2^endometrial, ^3^ovarian, and ^4^vulvar carcinomas. ^5^1 cervical and 5 endometrial samples had 2 *PIK3CA* mutations, and 11 endometrial samples had 2 *PTEN* mutations in the same tumour. One endometrial sample had 2 *CTNNB1* mutations and 1 cervical sample had 2 *PPP2R1A* mutations in the same tumour. Frequencies presented as N(%), where N represents the number of samples showing the mutation. Mutations that were included in the panel but were not detected are not shown.

The detected mutation frequencies were compared with the predicted numbers of mutations based on the frequencies reported in the COSMIC database [23] and corrected for the panel coverage (Table 4). *PIK3CA* mutations were detected twice as frequently as predicted in cervical cancer (*N* = 23 predicted and *N = *51 detected) and in endometrial cancer (*N* = 32 predicted and *N = *71 detected). *PTEN* mutations were also detected more frequently in endometrial cancer than predicted (*N = *35 predicted and *N = *104 detected). However, no *PTEN* mutations were detected in vulvar cancer although *N = *8 mutations were predicted [19].

**Table 4:** Coverage and frequencies of mutations in the gynaecologic-specific mutation panel.

	Cervical carcinoma *N = 205*						Endometrial carcinoma *N = 227*						Ovarian carcinoma *N = 89*						Vulvar carcinoma *N = 25*						Total Cohort *N = 546*					
	COSMIC^1^		GynCarta 2.0				COSMIC^1^		GynCarta 2.0				COSMIC^1^		GynCarta 2.0				COSMIC^1^		GynCarta 2.0				COSMIC^1^		GynCarta 2.0			
	Frequency	Percentage	% Coverage	*N e*xpected	*N* detected	Percentage	Frequency	Percentage	% Coverage	*N e*xpected	*N* detected	Percentage	Frequency	Percentage	% Coverage	*N e*xpected	*N* detected	Percentage	Frequency	Percentage	% Coverage	*N e*xpected	*N* detected	Percentage	Frequency	Percentage	% Coverage	*N e*xpected	*N* detected	Percentage
*BRAF*	6/434	1	0	0	0	0	33/2254	1	26	1	0	0	253/3398	7	95	6	0	0	-	-	-	-	0	0	292/6086	5	86	7	0	0
*CDKN2A*	23/248	9	8	2	5	2	13/427	3	33	2	0	0	63/1378	5	13	1	1	1	1/27	4	100	1	3	12	100/2080	5	16	6	9	2
*CTNNB1*	7/130	5	57	6	7	3	283/1309	22	91	45	33	15	105/1521	7	86	5	3	3	-	-	-	-	0	0	395/2960	13	88	56	43	8
*FBXW7*	1/12	8	0	0	3	1	33/307	11	22	5	12	5	6/882	<1	33	0	1	1	-	-	-	-	0	0	40/1201	3	27	5	16	3
*FGFR2*	2/58	3	0	0	1	<1	88/927	9	77	16	13	6	4/857	<1	50	0	1	1	-	-	-	-	0	0	94/1842	5	71	16	15	3
*FGFR3*	6/414	1	83	2	1	<1	2/262	1	0	0	0	0	0/792	0	-	-	0	0	-	-	-	-	0	0	8/1468	<1	83	2	1	<1
*FOXL2*	0/28	0	-	-	0	0	0/216	0	-	-	0	0	329/1794	18	100	16	0	0	-	-	-	-	0	0	329/2038	16	100	16	0	0
*HRAS*	15/215	7	87	12	0	0	0/528	0	-	-	0	0	0/731	0	-	-	0	0	0/13	0	-	-	2	8	15/2487	1	87	12	2	<1
*KRAS*	45/617	7	100	14	9	4	327/2578	14	99	32	39	17	522/4203	12	99	11	16	18	0/14	0	-	-	0	0	894/7412	12	99	57	64	12
*NRAS*	2/127	2	100	4	1	<1	11/548	2	100	5	6	3	3/780	<1	100	0	1	1	0/13	0	-	-	0	0	16/1468	1	100	9	8	1
*PIK3CA* *	39/332	12	94	23	51	24	562/2550	22	70	35	71	31	198/2366	8	85	6	10	11	-	-	-	-	1	4	799/5250	15	76	64	133	24
*PPP2R1A* *	2/14	14	0	0	8	3	76/645	12	72	20	18	8	36/1354	3	84	2	2	1	-	-	-	-	0	0	114/2013	6	75	22	28	5
*PTEN* *	20/406	5	29	3	5	2	824/2170	38	40	35	104	46	53/1487	4	40	1	4	3	5/8	63	54	8	0	0	906/4078	22	40	47	113	21

*The absolute number of mutations are reported; >1 mutation was detected in some tumours. COSMIC^1^ database accessed February 2013. ‘-’ indicates that data were not applicable.

Furthermore, no *BRAF* or *FOXL2* mutations were detected in this cohort, despite the high coverage of both genes by the panel. This could be explained by the fact that *FOXL2* is strongly associated with granulosa cell tumours of the ovary [30], a subtype of ovarian cancer that was excluded from our study cohort.

### GynCarta 2.0 can be used in differentiating tumour types

A visual illustration summarising the mutation frequencies in the different tumour types is depicted in figure 2. As shown in figure 2, gynaecological tumours show considerable overlap in somatic mutations, though tissue specific profiles can also be appreciated.

Endometrial cancers have the highest mutation frequency, with 78% of the samples carrying at least one mutation. As predicted, the most frequently mutated genes in gynaecological cancers are genes of the pAKT/mTOR pathway, but within this pathway, the mutational frequencies vary between tumour types. For ovarian cancer, *KRAS* is the most frequently mutated gene (18%), whereas *PIK3CA* is mostly affected in cervical cancer (24%) and *PTEN* in endometrial cancer (39%). Although the numbers of vulvar carcinomas included are small, vulvar cancer seems to have a different mutational spectrum as compared to other gynaecological malignancies with *CDKN2A* (12%) and *HRAS* (8%) most often affected.

An interesting difference can be observed when comparing *PIK3CA* distribution between cervical cancers and the other tumour types. In endometrial (and ovarian cancer), *PIK3CA* mutations are found most frequently on hotspots located on exon 9 and exon 20, with an even distribution between these exons (33% and 45%). In cervical cancer however, mutations almost exclusively occur on loci on exon 9 (47 out of 50 (94%) *PIK3CA* mutations). This clear difference (*p*<0.0001) can be used in clinical practice, when differentiating primary cervical cancer from primary endometrial cancer.

## Discussion

The demand for individualized cancer therapy has increased in recent years. New genotyping techniques allow tumours to be characterized based on their genomic profiles, which has revealed new targets for tumour-specific treatment, provided insights into tumour response to chemo- and radiotherapies, and helped predict patient outcome [3]–[6], [9], [12], [14]. Gynaecological malignancies account for 15–20% of all malignancies in women worldwide [7]. The clinical consequences of somatic mutations in various gynaecological malignancies are not yet fully understood. In the present study, we designed a panel that is highly specific for a broad range of gynaecological cancers, to investigated the tumour-specific mutation spectrum of 162 mutations of 13 genes. Using this panel, we found that in this series somatic mutations were present in 36% of all cervical carcinomas, in 78% of endometrial carcinomas, in 37% of ovarian carcinomas and in 20% of vulvar carcinomas.

Somatic mutation spectra were investigated previously in gynaecological cancers also using MALDI-TOF [17], [18], [22], [31]–[33]. However, most of those studies used generic cancer gene panels based on the reported frequencies in all solid tumours or used pre-existing panels that were designed for general oncology [17], [22], [31]–[33]. These pre-existing, commercially available panels are not adjusted to the field of gynaecological oncology, with the disadvantage of containing genes that are not involved in gynaecological cancers such as *FLT3* and *KIT*, or omitting genes that have shown to be involved relatively frequent in gynaecological cancer, such as *PIK3CA*. Therefore, we created a MALDI-TOF-based mutation panel designed specifically to detect a wide range of the most common hotspot mutations that have been reported in various types of gynaecological tumours. Similar mutation panels have been designed specifically for melanomas, colon carcinomas and non-small cell lung cancer [15], [16], [20]. By using a gynaecological specific panel, we studied only relevant mutations, including for example *PIK3CA* and *PPP2R1A* that are not incorporated in general panels such as the OncoCarta (Sequenom, Hamburg, Germany) and with a better and more specific coverage (for e.g *CTNNB1*). As a result, the reported frequencies of gene-involvement can differ substantially. For example, in our series of endometrial cancer, a *KRAS* mutation rate of 17% was detected. This is in contrast to the study of Cote et al [32] that, using a generic onco-panel, reports a *KRAS* mutation rate of only 1% in endometrial cancer. From other studies using different techniques, it is known that *KRAS* is mutated in 15–20% of all endometrial cancers [18], [34]. This example shows that the reliability of studies using a MALDI-TOF approach is seriously influenced by the choice and the extent of coverage of the genes incorporated in the panel.

Satisfactory coverage of the genes in our panel was achieved for the mutations we studied, and the mutation spectra generated in this study are thus a reliable representation of the mutation frequencies in gynaecological malignancies in the genes that are selected for this panel. However, some relevant genes, such as *TP53* and *ARID1a*, [8], [13], [34]–[39] were not included in our panel, because they did not fulfil the criterium of a “hotspot gene”. Both genes have mutations scattered widely throughout the gene and were therefore not suited for a MALDI-TOF approach. There are some loci in *TP53* and *ARID1a* that are more frequently mutated; however these cover no more than 20% of all its known mutations. Including some of these loci in our panel would underestimate the true mutational frequency of these genes in gynaecologic cancers. Their mutation frequencies could be studied better using other detection methods, such as Sanger sequencing or by next generation sequencing (NGS).

We did decide to include 22 assays for the tumour suppressor gene *PTEN*, resulting in a 40% coverage, which could be considered suboptimal using this approach. The mutation frequency reported here is therefore likely underestimating the true somatic mutational frequency of *PTEN*. Additionally, loss of PTEN can also be caused by other molecular alterations, such as LOH and promoter hypermethylation [40]. Therefore, the additional use of other techniques such as immunohistochemistry is advised to evaluate the true status of PTEN.

*CDKN2A* is not truly a hotspot mutated gene too, but it was added to the panel because of its high predicted relevance in vulvar cancer, and because we expected to obtain a fair coverage of the gene. Tumours included in the COSMIC database frequently show complete loss of, or large deletions in the *CDKN2A* gene, a type of mutation that is not easily detectable by MALDI-TOF mass spectrometry. However, since *CDKN2A* mutations in squamous cell carcinoma of the skin are reported to be more often point mutations than (large) deletions, we believe that adding *CDKN2A* point mutations to the panel can give valuable information, especially for vulvar cancer. Although numbers are very low, results from research on *CDKN2A* imutations in vulvar and penile squamous cell carcinoma strengthen this hypothesis [41].

*FBXW7* appears to have a low coverage by the panel, but this is influenced by the fact that it has been investigated and found to be mutated in relatively small numbers of gynaecological tumours. When considering the large numbers of available data from research in colon cancer, the expected coverage is approximately 35%.

Novel technologies such as next generation sequencing are able to detect mutations in multiple genes without preselecting and can therefore overcome the limitations of a mass-spectrometry approach. With NGS, complete genes of interest can be analysed and therefore all mutations will be found. However, bioinformatic analysis of the data produced by NGS can be challenging and is currently still in development. Additionally, differentiating between non-pathogenic somatic variants and pathogenic mutations can be time consuming and complex [42]. In comparison, MALDI-TOF data analysis is much more straightforward, particularly when analysing mutations with known clinical relevance. The panel we present here covers the most frequent mutations in gynaecological cancers, with a few exceptions. The mutation spectra we have detected are comparable to the spectra reported in NGS and exome sequencing studies that focus on gynaecological cancers [8], [43], [44]. Therefore, MALDI-TOF mass-spectrometry has potential for use in a clinical setting, to detect the mutational status of relevant genes in a fast and reliable way. Another clear advantage of mass spectrometry based mutation analysis is the flexibility to add and delete assays from a panel, as also shown in this report, so new insights or clinical demands can be adopted easily.

The somatic mutation landscape of gynaecologic cancers produced by this study (figure 2) and by publicly available mutation libraries show overlapping and distinguishing mutation profiles between gynaecologic tumours. Mutations in the PI3K/Akt-pathway are frequent and overlap, however some distinguishing mutations were identified. An example is the finding that *PIK3CA* exon 20 mutations only rarely occur in cervical cancer, whereas they are a frequent finding in endometrial cancers. This finding may be of value in a clinical setting, when there is uncertainty about the tumours primary origin, particularly in cervical adenocarcinomas that are HPV negative and located in the low uterine segment of the uterus. It illustrates that somatic mutational information may be useful for classifying tumours [45].

Somatic mutation profiling can also reveal new insights into tumour types that are not well characterised yet, such as vulvar cancer. Vulvar cancer is a rare disease that can arise through an HPV-dependent or an HPV-independent pathway. The carcinogenesis of HPV-independent vulvar carcinomas is largely unknown. In the present study, 25 vulvar carcinomas (of which 19 HPV-negative tumours) were analysed, and one *PIK3CA*, 3 *CDKN2A*, and 2 *HRAS* mutations were detected in the HPV-negative carcinomas. No mutations were detected in any of the 13 investigated genes in the 6 HPV-positive tumours. The mutation spectrum of vulvar cancer seems different from the spectrum of other gynaecological cancers, but shows similarities to the mutation spectrum of squamous cell carcinoma's of the head and neck [46], a tumour type that shares morphological and etiological characteristics with vulvar squamous cell carcinoma. The fact that vulvar cancer does not arise in Mullarian originated structures, as the other three tumour types in this study do, could also be an explanation for the differences in the spectrum that we have detected. The results of our study prompt further investigation of the roles of *HRAS* and *CDKN2A* in vulvar cancer.

In conclusion, we designed, validated and used a novel mass spectrometry-based mutation panel to identify somatic mutations in a large cohort of gynaecological malignancies. We have shown that this new panel is reproducible, high-throughput, and suitable for low quality and quantity DNA from FFPE samples. Our data support the potential for somatic mutation profiles as a tool to classify tumour types within the gynaecological tract. Furthermore, our results revealed that the PI3K-Akt signalling pathway is most prominently affected in gynaecological malignancies, justifying further investigation of *PI3K*/AKT/mTOR targeting therapies in gynaecological oncology. Future studies are required to determine whether this panel can be used to predict effective individualized, tumour-specific, and targeted treatment approaches.
